# Sodium Reduction in Processed Foods in Brazil: Analysis of Food Categories and Voluntary Targets from 2011 to 2017

**DOI:** 10.3390/nu9070742

**Published:** 2017-07-12

**Authors:** Eduardo A. F. Nilson, Ana M. Spaniol, Vivian S. S. Gonçalves, Iracema Moura, Sara A. Silva, Mary L’Abbé, Patricia C. Jaime

**Affiliations:** 1 Ministry of Health of Brazil, Brasilia 70058-900, Brazil; ana.spaniol@saude.gov.br (A.M.S.); vivian.goncalves@saude.gov.br (V.S.S.G.); iracema.moura@saude.gov.br (I.M.); sara.silva@saude.gov.br (S.A.S.); 2 Global Health and Sustainability Program, University of Sao Paulo, Sao Paulo 01255-001, Brazil; 3 University of Toronto, Toronto, ON M5S 2E8, Canada; Mary.Labbe@utoronto.ca; 4 Department of Nutrition, University of Sao Paulo, Sao Paulo 01255-001, Brazil; constant@usp.br

**Keywords:** sodium, processed foods, hypertension, cardiovascular disease, food reformulation

## Abstract

Non-communicable diseases, including cardiovascular diseases, are responsible for over 70% of deaths in Brazil. Currently, over 25% of Brazilian adults are diagnosed as hypertensive; overall, current dietary sodium intake in Brazil (4700 mg/person) is over twice the international recommendations, and 70–90% of adolescents and adults consume excessive sodium. National sodium reduction strategies consider the main dietary sources of sodium to be added salt to foods, foods consumed outside of the household, and sodium in processed foods. The national voluntary strategy for sodium reduction in priority food categories has been continuously monitored over a 6-year period (2011–2017) and there was a significant 8–34% reduction in the average sodium content of over half food categories. Different food categories have undergone differing reductions in sodium over time, aiding gradual biannual targets to allow industries to develop new technologies and consumers to adapt to foods with less salt. By 2017, most products of all food categories had met the regional targets proposed by the Pan American Health Organization, showing that voluntary sodium reduction strategies can potentially contribute to food reformulation. Nevertheless, regulatory approaches may still be necessary in the future in order to reach all food producers and to allow stronger enforcement to meet more stringent regional targets.

## 1. Introduction

In most countries in the world, there is excessive dietary sodium consumption within the population, which is an important risk factor for the development of hypertension and cardiovascular disease [1]. Accordingly, sodium reduction was prioritized in the United Nations’ Global Action Plan for the Prevention and Control of Noncommunicable Diseases (NCD), and the World Health Organization (WHO) has defined a 30% relative reduction in mean population intake of salt/sodium as a global voluntary target for 2025 [2]. The Pan American Health Organization (PAHO) has followed global priorities by urging governments to commit to the global NCD targets and supporting countries of the Americas in reducing dietary sodium to less than 2000 mg per person by 2020 [3].

Diet is an important risk factor for NCDs. Dietary sodium reduction is a modifiable risk factor for hypertension and cardiovascular disease and a highly cost-effective strategy (“best-buy”) in non-communicable disease prevention as per the WHO [4]. It has been projected that a 10% worldwide reduction in sodium consumption over 10 years would avert millions of disability-adjusted life years (DALYs) and hundreds of thousands of deaths related to cardiovascular diseases [5]. The epidemiologic and economic burdens of NCDs in Brazil are also substantial. Non-communicable diseases are the main cause of mortality in the country (72.8% of deaths) and cardiovascular diseases have been the leading cause of death since the 1960s, accounting for 20% of total deaths in 2013 [6,7]. The prevalence of diagnosed hypertension in Brazilian adults has increased by over 14%, from 22.5 to 25.7% over a ten-year period (2006 to 2016) according to the 2016 National Telephone Survey (Vigitel) [8]. The overall hospitalization costs due to cardiovascular diseases have increased approximately 40% from 2008 to 2016 (from the equivalent of US$448 million to US$741 million), according to the National Healthcare Expenditure Database (Sistema de Informações Hospitalares do Sistema Único de Saúde/SIH-SUS), which covers more than 70% of all hospital admissions in Brazil.

Over the last decades, food consumption has been changing rapidly in Brazil and processed foods are replacing staple foods in diets [9]. The participation of processed foods in dietary sodium is continuously increasing and excessive sodium consumption has been directly related to the share of processed foods in the diet [10], although salt and salt-based condiments added to foods are the main sodium source in the diet [11].

The need for sodium reduction in processed foods is also supported by recent data on nutritional profiling in Brazil, which evidences the high sodium content of most processed foods [12] and even of foods targeted at children [13]. The average sodium consumption of Brazilians (4700 mg/day) is over twice the World Health Organization maximum recommendation and 70–90% of adolescents and adults consume excessive dietary sodium (over 2000 mg/day) [14]. Nevertheless, in the 2013 National Health Survey, people were asked about their self-perception of salt/sodium consumption, and only 14% of the adults considered their consumption as high and over 80% of the population perceived it as adequate or low [15].

In the last decade, national sodium reduction policies have been implemented by many regions of the world, including multi-component strategies and individual strategies such as mandatory and voluntary food reformulation, taxation of unhealthy foods, school interventions, dietary advice, community-based counseling, and nutritional labeling. Recent evidence suggests that population-wide policies and comprehensive strategies involving food reformulation, food labeling, media campaigns and mandatory reformulation may achieve larger reductions in salt consumption than focused interventions [16,17].

A systematic review of progress in sodium reduction policies in processed foods in 75 countries of all regions in the world showed that voluntary agreements with food industries are more commonly implemented (36 countries), while only nine countries have set mandatory limits for sodium in processed foods [18]. 

In general, regulatory approaches are able to be enforced more effectively, but it is more difficult for them to be approved and updated regularly. In contrast, voluntary strategies are more easily implemented and adjusted over time, but rely on industry commitment and strong monitoring to achieve changes in the nutritional profile of foods [18,19].

National sodium reduction strategies have been monitored by assessing compliance with sodium reduction targets (voluntary or mandatory) and the changes in sodium levels in foods, mostly using commercial label data.

In the United Kingdom, where a long-term voluntary sodium reduction program coupled with public awareness campaigns has been in place since 2006, a reduction in sodium levels in processed foods has already impacted the overall sodium intake of the population [20]. Voluntary strategies in Australia and New Zealand have also shown a reduction in mean sodium levels in several food categories, both through food label collection [21] and analytical data [22].

In Argentina, mandatory maximum levels for meat and farinaceous products as well as soups and dressings were set in 2013, following previous voluntary agreements. Currently, most of the food groups included in the law already have sodium content within the maximum limits, so further reductions of the existing limits are required, and more food categories should be included in the law [23]. In South Africa, industries are rapidly meeting the mandatory limits for sodium content in processed foods and, one year after the introduction of the national legislation, two-thirds of targeted foods have met the established limits and many others are close to meeting the legislated requirements [24]. The Brazilian Dietary Sodium Reduction Plan takes into account the multiple dietary sources of sodium and the needs of different population groups, and involves consumer education, healthy diet promotion, processed food reformulation, health promotion in school and work settings, food regulation, and healthcare organization [25].

The Dietary Guidelines for the Brazilian Population are the major tool for food and nutrition education and state that salt should only be used in small amounts when cooking and consuming foods and that so called ultra-processed foods, commonly rich in sodium, sugars, and fats, should be avoided [26]. 

The food reformulation strategies in Brazil have been based on voluntary agreements with the Brazilian Association of Food Industries (ABIA), which accounts for over 70% of the processed food market in the country. Targets were set in order to represent gradual but meaningful reductions in the maximum sodium content of packaged foods through biannual targets for the food categories that most contribute to sodium intake [27], according to national household budget surveys [28]. The Pan American Health Organization has supported sodium reduction policies in the Americas and proposed inaugural regional targets for several food categories in 2014 (breads, cakes, cookies and biscuits, pastas, dairy spreads, breakfast cereals and mayonnaise). These targets were based on the national targets for sodium reduction in the region, either voluntary or regulatory, as in Argentina, Brazil, Canada, Chile and the United States. The maximum values set by the PAHO consist of a general target for each food category. A more stringent target is also proposed, based on the lowest targets in the region, in order to assist countries in starting their national sodium reduction policies and to improve the ongoing policies [29].

Thus the main purpose of this study was to follow-up the changes in sodium content of the main food categories with voluntary sodium reduction targets in Brazil from 2011 to 2017 and to compare the sodium content of food categories in Brazil to the regional targets proposed by the PAHO.

## 2. Materials and Methods

### 2.1. Product Selection Criteria

The food categories that were analyzed in this study include breads, cakes and cake mixes, pastas, snacks, mayonnaise, dairy spreads, margarines, salt-based condiments, mozzarella cheese, biscuits, cookies and crackers, and breakfast cereals, which were prioritized for sodium reduction with food industries.

Some food categories were divided into subcategories for specific sodium reduction targets because of technical justifications by food industries when targets were established. Breads include sliced bread and buns, cakes are subdivided into those with or without filling, cake mixes may be aerated or creamy, snacks include extruded corn snacks and potato chips, and condiments include bouillons, paste and rice condiments [30,31,32,33]. Prior to data collection, food companies that voluntarily committed to the national sodium reduction targets and their products were listed by the Brazilian Association of Food Industries.

The food subcategories that were included in this study were required to have completed two monitoring cycles, considering the baseline of target setting (in 2011) and data collection rounds every two or three years (2013–2014 and 2017). Food categories that did not complete at least 4 years of target implementation and products belonging to companies that are not members of ABIA were not included in the analysis.

### 2.2. Data Collection

Data on sodium content were directly obtained from the mandatory food label information of products, mainly from the official company websites. All nutritional information from products that was not available in the websites was collected through the companies’ customer services. The products also needed to be available on the market at the time of data collection according to the information provided by ABIA, and in the case of different package sizes for the same product, only one entry was considered. Baseline and follow-up data consisted of records of the manufacturer, brand and commercial product name, as well as the sodium content per sizing and adjusted per 100 g. Data were independently verified for outliers, missing values and data entry accuracy by two study personnel and queries and discrepancies were reviewed from the websites and followed-up directly with the food industries and their association.

### 2.3. Statistical Analysis

Firstly, data were independently verified for outliers, missing values and data entry accuracy by two study personnel, and queries and discrepancies were reviewed from the websites and directly with the food industries and ABIA when necessary.

The sodium content of all food subcategories in 2017 was directly compared to the PAHO regional targets (lowest and most stringent). Some food subcategories were grouped in order to be compared to the regional food categories (breads, pasta, snacks, mayonnaise, butter/dairy spread, condiments, cookies and sweet biscuits, savory biscuits and crackers, and breakfast cereals) and food categories which did not have corresponding regional targets were compared to the 2016 national targets [29].

For all food categories studied, descriptive statistics were calculated including the total of products for each category, measures of central tendency and dispersion (means and medians, as well as their respective standard deviations and maximum and minimum sodium levels) in 2011, 2013–2014 and 2017. Then, the sodium content of each food category was calculated at the same time points based on variable distribution to verify normality through the Shapiro–Wilk test. After that, we investigated the significance of the differences in sodium content at each data collection point through the Kruskal–Wallis test, a non-parametric test, because the data studied did not meet the normality criteria. For the food categories that presented statistical significance, we conducted the Dunn’s test to verify statistical difference in each time period (2011 to 2013–2014, 2013–2014 to 2017 and 2011 to 2017). All analyses considered statistical significance as *p* < 0.05. 

Additionally, data were plotted in distribution graphs of sodium values from baseline to the second round of monitoring, displaying the interquartile ranges.

All statistical analyses were conducted using Stata 12 (Stata Corp, College Station, TX, USA).

## 3. Results

### 3.1. Proportion of Products Meeting the PAHO Regional Targets

After removing duplicates and products with ineligible or insufficient information on nutritional composition, we analyzed a total of 1067 products at baseline, 1288 products in the first data collection cycle (2013–2014) and 981 products in the second data collection cycle (2017). According to the Brazilian Association of Food Industries, the different number of food products within each time period is due to discontinuity and replacement of products over time.

The sodium content of Brazilian processed foods analyzed in 2017 indicates that in over half of the food subcategories 100% of the products met these targets, and in all except one food subcategory (corn snacks), over 85% of products met the regional targets for 2017. 

Considering the more stringent maximum values set by the PAHO for the region, the targets were met by over 70% of the products in half of the food subcategories, and only breakfast cereals had all products meet these maximum values. In addition, nine food subcategories had a less than 50% compliance in their products when compared to the more stringent targets, of which three subcategories had less than 10% of the products meet these targets, as shown in Table 1.

### 3.2. Analysis of Mean and Median Sodium Content for Each Food Category over Time

The mean and median sodium content of all 20 food subcategories, along with their standard deviations and minimum and maximum values, were evaluated at the baseline of the target setting (2011), in 2013–2014, and in 2017.

Sodium changes varied between food subcategories, considering the measures of central tendency of sodium content of food products at baseline and at each data collection point (Table 2). Most food subcategories (except for corn snacks and mozzarella cheese) continually reduced both sodium means and medians over time, and statistically significant reductions were found for 65% of the subcategories from 2011 to 2017.

**Figure 1:**
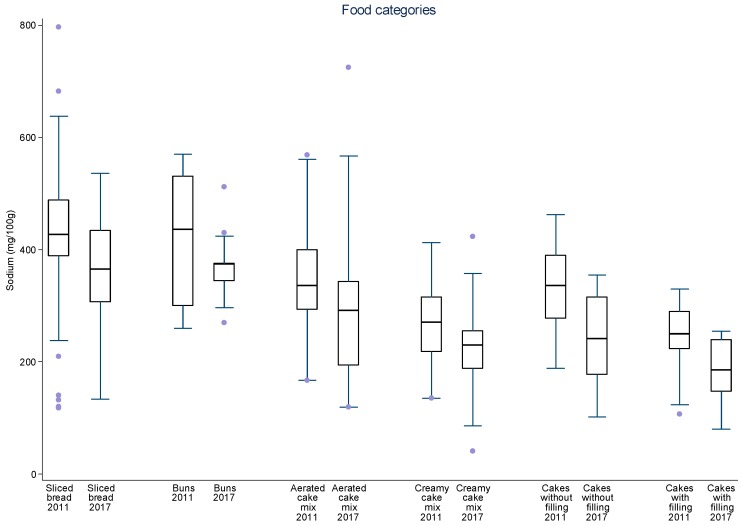
Distribution of sodium values from baseline to the second data collection for sliced bread, buns, aerated cake mixes, creamy cake mixes, cakes without filling and cakes with filling. The box displays the interquartile range and the median value is marked as a line within the box. The lines extending above and below the box indicate the most extreme value within the 75th percentile + 1.5*x* (interquartile range) and the 25th percentile − 1.5*x* (interquartile range), and additional values outside of this range are marked as grey circles.

**Figure 2:**
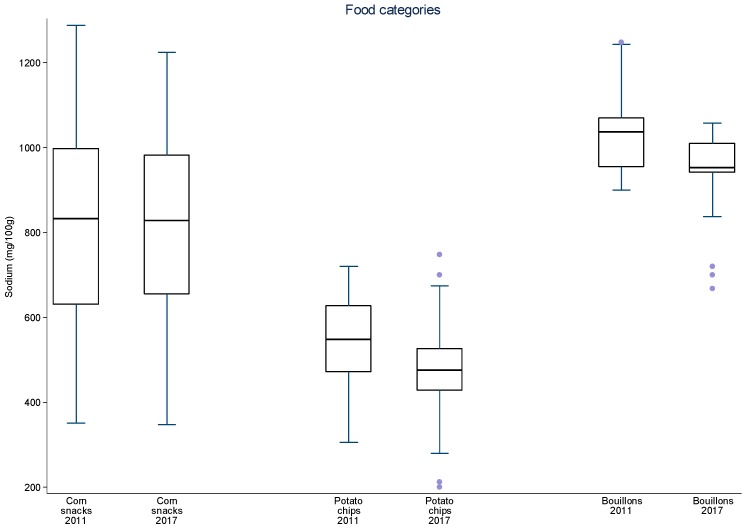
Distribution of sodium values from baseline to the second data collection for corn snacks, potato chips and bouillons. The box displays the interquartile range and the median value is marked as a line within the box. The lines extending above and below the box indicate the most extreme value within the 75th percentile + 1.5*x* (interquartile range) and the 25th percentile − 1.5*x* (interquartile range), and additional values outside of this range are marked as grey circles.

**Figure 3:**
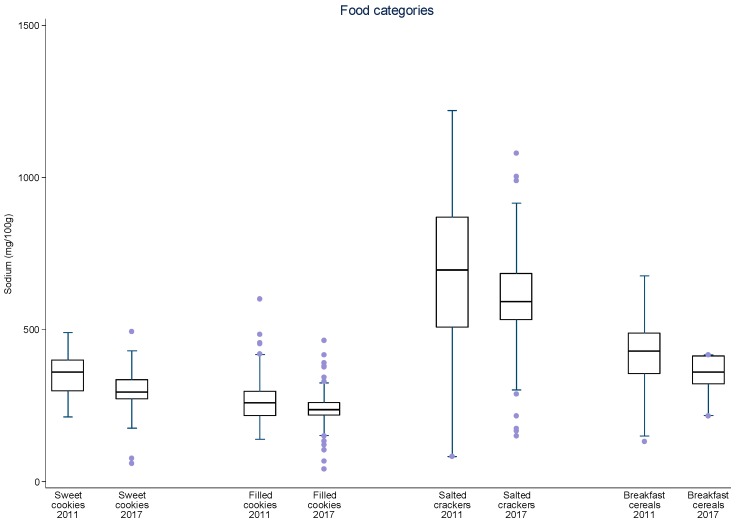
Distribution of sodium values from baseline to the second data collection for sweet cookies, filled cookies, salted crackers and breakfast cereals. The box displays the interquartile range and the median value is marked as a line within the box. The lines extending above and below the box indicate the most extreme value within the 75th percentile + 1.5*x* (interquartile range) and the 25th percentile − 1.5*x* (interquartile range), and additional values outside of this range are marked as grey circles.

**Figure 4:**
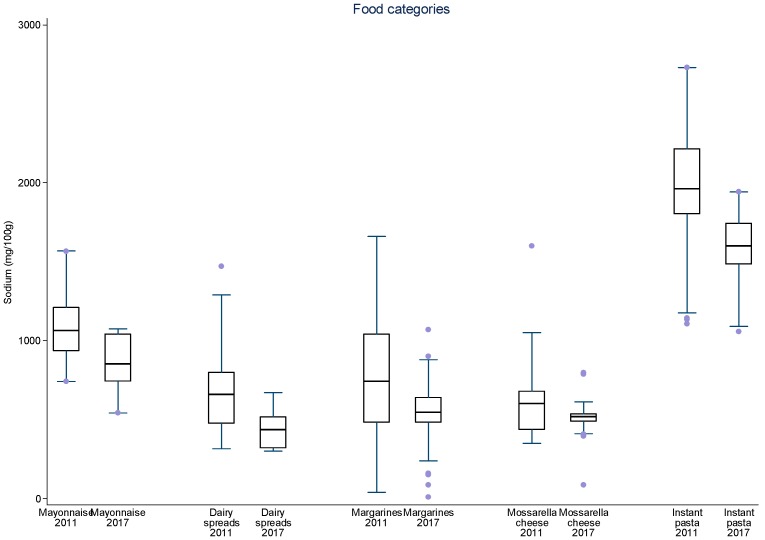
Distribution of sodium values from baseline to the second data collection for mayonnaise, dairy spreads, margarine, mozzarella cheese and instant pastas. The box displays the interquartile range and the median value is marked as a line within the box. The lines extending above and below the box indicate the most extreme value within the 75th percentile + 1.5*x* (interquartile range) and the 25th percentile − 1.5*x* (interquartile range), and additional values outside of this range are marked as grey circles.

**Figure 5:**
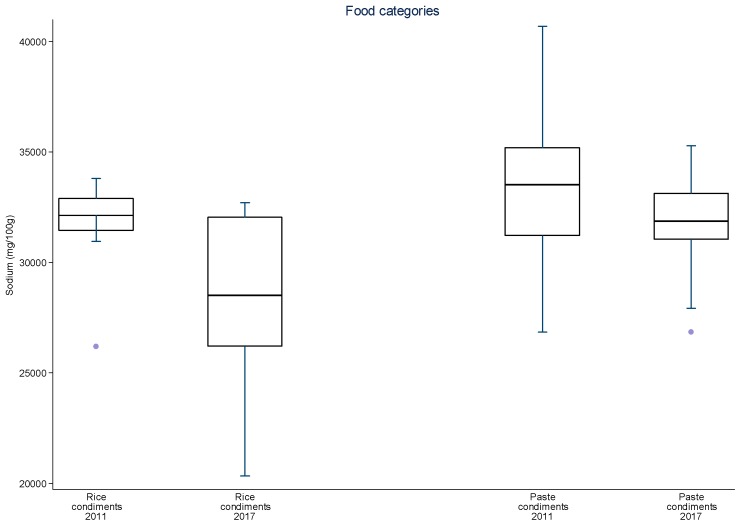
Distribution of sodium values from baseline to the second data collection for rice and paste condiments. The box displays the interquartile range and the median value is marked as a line within the box. The lines extending above and below the box indicate the most extreme value within the 75th percentile + 1.5*x* (interquartile range) and the 25th percentile − 1.5*x* (interquartile range), and additional values outside of this range are marked as grey circles.

For sliced bread, salted crackers, sweet biscuits, filled cookies, instant pastas, mayonnaise, cakes with and without filling, aerated and creamy cake mixes, margarines, dairy spreads and bouillons, there was a significant 8–34% reduction in mean sodium content between 2011 and 2017 (Table 3). The greatest sodium reductions occurred for cakes (25.7 to 28.0%), margarines (26.4%) and dairy spreads (28.0%). The speed of sodium reduction varied among the food subcategories between each monitoring cycle but most subcategories evidenced continual reduction of mean sodium content. Significant reductions in mean sodium content were achieved in nine subcategories between 2011 and 2013–2014 and in six subcategories in 2013–2014 and 2017. Four subcategories significantly reduced their mean sodium content in both periods (aerated cake mixes, cakes with and without filling and dairy spreads). Despite the reduction in mean sodium content for most subcategories in both periods, for mozzarella cheese and corn snacks, an initial reduction was followed an increase in overall mean sodium content. At this point, other studies pairing products from each period may for allow a better analysis about these different rates of sodium reduction. The distribution of sodium content varied highly between 2011 and 2017 amongst food subcategories and between time periods (Figure 1, Figure 2, Figure 3, Figure 4 and Figure 5). The variability in sodium content within food subcategories declined for 70% (14) of the subcategories, converging towards the median sodium content (sliced breads, buns, creamy cake mixes, potato chips, bouillons, sweet cookies, filed cookies, salted crackers, breakfast cereals, dairy spreads, margarines, mozzarella cheese, instant pasta and paste condiments). The upper values of the interquartile ranges of all and the lower limits were also reduced for most categories.

## 4. Discussion

Brazil initiated the reduction of the sodium content of packaged foods in 2011 and has set biannual voluntary targets for food industries with respect to the maximum levels of sodium for the categories that contribute to over 90% of the sodium from industrialized foods. Therefore, the Brazilian sodium reduction strategy for processed foods relies on the commitment of the major food industry association in the country.

The extent of the impact of sodium reduction in processed foods has been questioned [34], but it must be considered part of the reduction of dietary sodium in Brazil and accurate results depend on updated food composition data to follow food reformulation. It is also important to consider that sodium reduction strategies include ready-to-eat foods and processed culinary ingredients, as bouillons and other salt-based condiments [35].

Reducing the dietary sodium intake of the Brazilian population from its current levels to the intended 2 g/day will require a combination of strategies to address all dietary sources of sodium. These strategies include the promotion of healthy diets (including awareness on the risks of excessive dietary sodium and the reduction of discretionary salt use), the promotion of healthy environments (especially schools, including restrictions to unhealthy foods), food regulation (for example front of pack information and other improvements in food labeling) and salt reduction in food services and restaurants.

Although national and international experiences have generally focused on single-nutrient approaches (especially for sodium), recent discussion of multi-nutrient profiling systems may provide broader reformulation strategies and more benefits to public health in the future through combined reduction of sodium, sugars and fats [36].

The data here presented provide an evaluation of the changes in sodium content of processed foods through voluntary sodium reduction targets in Brazil. Our results show overall progress in sodium reduction in most food subcategories, although it is apparent that some subcategories may not achieve the targets or may slow their reductions in the long term.

These results suggest that reformulation targets for sodium affect the upper limit of sodium content of food subcategories, as intended, and also induce changes in the subcategories as a whole by reducing the mean and median sodium content and by affecting the distribution of sodium content within each category.

The variation of sodium content in processed foods over four to six years in Brazil also suggests there may be category-specific issues and challenges that influence the extent of sodium reduction over time and amongst food categories. Nevertheless, it is likely that gradual reductions in sodium content allow food industries to develop the alternatives to reduce sodium more significantly and for consumers to adapt to foods with less sodium.

Sodium is important in processed foods for microbiologic protection, shelf life, sensorial characteristics (such as taste and crustiness), and performance of industrial processes, so these functional roles must be carefully considered in food reformulation [37]. Most food categories have met the PAHO regional targets for sodium reduction, although, considering the most stringent targets, many categories still need further sodium reduction.

Based on the comparison of Brazilian targets and the regional PAHO targets, it is also likely that the list of regional targets should be expanded and the existing inaugural targets may also need to be updated in order to advance in sodium reduction in the Americas.

These results contribute to the building of knowledge on voluntary and regulatory approaches to sodium reduction in packaged foods, such as the adoption of regulatory sodium targets by Argentina [38] and South Africa [24], while voluntary agreements with food industries have been adopted more frequently, based on successful experiences such as the one in the United Kingdom [38]. 

A key strength of this study is the completeness of data collection through accurate, updated and representative nutritional composition data and collection in food company websites. These data sources also allow systematic rounds of data collection for new assessments, including the introduction of new lower-sodium products and the discontinuity of other high-sodium products.

Our study also had several weaknesses. Firstly, food categories that had targets set after 2014 (meat products and soups) could not be included in this analysis because they did not complete two biannual rounds of data collection. Secondly, data collection and analysis only encompassed the products of companies that belonged to the Brazilian Association of Food Industries (ABIA) and did not include breads, cakes and other products from bakeries. Thirdly, we were also unable to assess food composition data using additional sources such as laboratory analysis, so the integrity of our nutritional data depended on the accuracy of food label information and the completeness and regular update of food product information on food company websites. Administrative reports by the National Health Surveillance Agency (ANVISA) suggest that food label information is generally accurate and reliable. Fourthly, all analyzes are based on simple means of sodium content, which do not consider the market share of each product. Sales-adjusted means, as used in Canada, the United States and the United Kingdom, express the actual contribution of each product to sodium consumption, although there is a reliance on access to very expensive market databases.

Future studies will allow a more complete understanding of the long-term impacts of voluntary strategies in Brazil, and assess the impact of these reductions on morbidity, mortality and costs of hypertension and cardiovascular disease and subsidized policy improvement. For example, in Argentina modeling studies of sodium reduction scenarios have contributed to the transition from voluntary to regulatory targets for sodium reduction [22].

## 5. Conclusions

The data here presented provide evidence that the voluntary approach to setting sodium reduction targets in Brazil is leading to a gradual reduction of sodium content in most food categories over time, and that these same monitoring results can be helpful for adjusting targets in the future in order to achieve maximum sodium reductions in 2020 or later. The continuous monitoring process to this point has revealed impacts comparable to regional references as well as the PAHO targets, although stronger enforcement by regulatory targets may be needed in the future with the help of policy-makers, health authorities and civil society in order to reach the overall food market and apply more stringent limits to sodium content in packaged foods. 

## Figures and Tables

**Table 1:** Proportion of food categories and subcategories that met the initial and the lower inaugural regional sodium reduction set by the Pan American Health Organization.

Food Categories and Subcategories		Regional Target	% Products That Have Met the Regional Targets (2017)	Lower Target	% Products That Have Met the Lower Regional Targets (2017)
Americas	Brazil	mg/100 g	%	mg/100 g	%
Breads	Sliced bread (*n* = 82)	600	100.0	400	59.8
	Buns (*n* = 11)		100.0		81.8
Cakes	Aerated cake mixes (*n* = 135)	400	90.4	205	25.9
	Creamy cake mixes (*n* = 24)		91.7		45.8
	Cakes without filling (*n* = 68)		100.0		36.8
	Cakes with filling (*n* = 48)		100.0		54.2
Shelf-stable pasta and noodles (dry, uncooked)	Instant pasta (*n* = 87)	1921	98.9	1333	10.3
Snacks	Corn snacks (*n* = 39)	900	53.8	530	10.2
	Potato chips (*n* = 29)		100.0		75.9
Mayonnaise	Mayonnaise (*n* = 28)	1050	85.7	670	17.9
Butter/dairy spread	Dairy spread (*n* = 45)	800	100.0	500	73.3
	Margarines (*n* = 46)		95.7		28.3
Cheese *	Mozzarella cheese (*n* = 28)	559	89.3	512 ^b^	50.0
Condiments	Rice condiments (*n* = 5)	33,100	100.0	9100	0.0
	Bouillon cubes and powders (*n =* 35) **	1025	97.1	900	11.4
	Paste condiments * (*n* = 14)	37,901	100.0	33,134	78.6
Cookies and sweet biscuits	Sweet biscuits (*n* = 52)	485	99.8	265	21.2
	Filled cookies (*n* = 185)		100.0		86.5
Savory biscuits and crackers	Salted crackers (*n* = 84)	1340	100.0	700	91.7
Breakfast cereals	Breakfast cereals (*n* = 15)	630	100.0	500	100.0

* National targets (food categories with no regional targets). ** Adjusted to portion size (5 g) according to Brazilian regulation.

**Table 2:** Sodium content of food subcategories at baseline and at the first and second monitoring cycles, Brazil 2011–2017.

Food Categories	*n*	Sodium 2011 (mg/100 g)		*n*	Sodium 2013–2014 (mg/100 g)		*n*	Sodium 2017 (mg/100 g)		*p* *
		Mean ± SD	Median (Min–Max)		Mean ± SD	Median (Min–Max)		Mean ± SD	Median (Min–Max)	
Sliced bread	117	426.5 ± 107.1 ^a,b^	432.0 (118.0−796.0)	87	380.3 ± 122.1 ^a^	380.0 (126.0−870.0)	82	365.0±87.6 ^b^	380.0 (134.0−536.0)	<0.001
Buns	9	436.1 ± 121.4	462.0 (260.0−570.0)	8	388.5 ± 74.4	415.0 (270.0−462.0)	11	374.4 ± 59.4	372.0 (270.0−512.0)	0.359
Aerated cake mixes	125	372.3 ± 173.4 ^a,b^	314.0 (166.7−1111.5)	201	309.6 ± 69.2 ^a,c^	327.0 (117.0−474.0)	135	291.6 ± 92.6 ^b,c^	293 (119.6−724.3)	<0.001
Creamy cake mixes	24	270.7 ± 75.6 ^a^	280.0 (135.1−412.0)	33	250.5 ± 44.6	251.0 (69.0−333.0)	24	229.6 ± 82.1 ^a^	226.2 (40.7−422.9)	0.047
Cakes without filling	64	335.7 ± 66.7 ^a,b^	355.0 (188.3−462.9)	69	281.0 ± 85.9 ^a,c^	300.0 (117.0−398.3)	68	241.1 ± 74.9 ^b,c^	250.0 (101.7−355.0)	<0.001
Cakes with filling	41	249.9 ± 51.4 ^a,b^	240.0 (107.0−330.0)	68	212.3 ± 47.0 ^a,c^	224.0 (108.3−330.0)	48	185.8 ± 55.0 ^b,c^	200.0 (80.0−255.0)	<0.001
Instant pastas	90	1960.0 ± 384.5 ^a,b^	1993.5 (1104.9−2729.1)	97	1662.3 ± 265.7 ^a^	1670.0 (1057.5−2548.6)	87	1598.6 ± 189.6 ^b^	1607.1 (1057.5−2548.6)	<0.001
Corn snacks	25	831.9 ± 226.1	840.0 (351.0−1288.0)	39	753.9 ± 140.1	756.0 (352.0−1032.0)	40	827.4 ± 242.8	884.0 (348.0−1224.0)	0.067
Potato chips	22	547.6 ± 123.6	598.0 (305.0−720.0)	28	513.3 ± 130.7	516.0 (276.0−700.0)	30	475.4 ± 137.9	507.3 (200.0−748.0)	0.237
Mayonnaise	31	1063.3 ± 198.2 ^a,b^	1058.3 (741.7−1566.7)	41	891.3 ± 157.9 ^a^	925.0 (400.0−1075.0)	29	852.7 ± 194.9 ^b^	933.3 (541.7−1075.0)	<0.001
Dairy spreads	80	659.5 ± 248.4 ^a,b^	596.7 (314.0−1470.0)	80	524.4 ± 188.2 ^a,c^	468.3 (300.0−1100.0)	45	434.5 ± 110.3 ^b,c^	410.0 (300.0−670.0)	<0.001
Margarines	94	739.9 ± 363.6 ^a^	730.0 (40.0−1660.0)	84	689.8 ± 351.4 ^b^	710.0 (0.0−1660.0)	46	544.3 ± 207.3 ^a,b^	600.0 (10.0−1070.0)	<0.001
Mozzarella cheese	26	600.2 ± 363.6 ^a^	540.0 (350.0−160.0)	51	461.2 ± 132.2 ^a^	486.7 (87.0−786.7)	28	517.2 ± 131.5	526.7 (86.7−796.7.0)	0.039
Rice condiments	5	31,425.1 ± 3009.7	32,120.0 (26.186.0−33,800.0)	4	29,530.0 ± 6140.7	32,370.0 (20,340.0−33,040.0)	5	28,505.1 ± 5237.6	31,260.0 (20,340.0−32,700.0)	0.325
Bouillon cubes and powders	41	1035.9 ± 94.4 ^a^	1015.0 (900.0−1247.0)	26	985.2 ± 105.8 ^b^	1019.0 (705.0−1183.0)	35	952.1 ± 88.2 ^a,b^	967.0 (668.0−1057.0)	0.003
Paste condiments	14	33,494.5 ± 4054.4	33,450.0 (26,840.0−40,700.0)	14	32,900.0 ± 3173.6	33,850.0 (26,840.0−37,280.0)	14	31,845.7 ± 2615.9	32,220.0 (26,840.0−35,280.0)	0.303
Sweet biscuits	17	359.2 ± 81.3 ^a,b^	386.7 (213.3−490.0)	45	318.2 ± 50.3 ^a^	317.0 (236.7−416.0)	52	293.9 ± 72.4 ^b^	306.7 (60.0−493.3)	0.019
Filled cookies	176	259.5 ± 66.0 ^a,b^	251.7 (140.0−600.0)	198	242.6 ± 48.9 ^a^	243.0 (127.0−390.0)	185	235.5 ± 57.3 ^b^	240.0 (41.7−463.3)	0.006
Salted crackers	39	695.8 ± 260.8 ^b^	686.7 (83.3−1220.0)	94	660.4 ± 147.1 ^c^	633.0 (350.0−923.0)	84	590.9 ± 163.4 ^b,c^	626.7 (150.0−1080.0)	0.031
Breakfast cereals	27	428.9 ± 141.8	430.0 (132.0−676.7)	21	406.7 ± 129.9	392.5 (195.0−679.3)	15	359.2 ± 69.5	390.0 (216.7−416.7)	0.209

* Kruskal–Wallis Test; ^a, b, c^ Dunn's Test: same letters in the same lines = *p* < 0.05.

**Table 3:** Reduction in mean sodium content of food subcategories, Brazil 2011−2017.

Food Categories	% Reduction in Mean Sodium		
	2011−2013/14	2013/14−2017	2011−2017
Loaf bread	10.8 *	3.9	14.3 *
Buns	11.0	3.6	14.2
Aerated cake mixes	16.9 *	5.8 *	21.8 *
Creamy cake mixes	7.4	8.4	15.2 *
Cakes without filling	16.1 *	14.2 *	28.0 *
Cakes with filling	14.8 *	12.7 *	25.7 *
Instant pastas	15.2 *	3.8	18.5 *
Corn snacks	9.4	−9.8	0.5
Potato chips	6.2	7.4	13.2
Mayonnaise	16.2 *	4.4	19.8 *
Dairy spreads	20.5 *	17.1 *	34.1 *
Margarines	6.8	21.0 *	26.4 *
Mozzarella cheese	23.2 *	−12.1	13.8
Rice condiments	6.0	3.5	9.3
Bouillon cubes and powders	4.8	3.3 *	8.0 *
Paste condiments	1.8	3.2	4.9
Sweet biscuits	11.4 *	7.9	18.4 *
Filled cookies	6.6 *	2.9	9.3 *
Salted crackers	5.0	10.4 *	15.0 *
Breakfast cereals	5.4	11.6	16.3

* *p* < 0.05.
